# Sentiment Analysis in Healthcare: A Comparison of VADER, BERT, and Flair NLP Models on Patient Reviews of Pain Management Physicians

**DOI:** 10.7759/cureus.88902

**Published:** 2025-07-28

**Authors:** Ishan Aggarwal, Stanley Joseph, Nikhil Jaganathan, Akshar Patel, Vikas Kumar, Mallikarjuna Devarapalli

**Affiliations:** 1 Anesthesiology and Perioperative Medicine, Medical College of Georgia, Augusta University, Augusta, USA

**Keywords:** ai in healthcare, bert, bias in ai algorithms, emotion analysis, flair, natural language processing (nlp), patient reported experience measures, patient-reported outcome, sentiment analysis

## Abstract

Natural language processing (NLP) has become an essential tool in healthcare, enabling sentiment analysis to extract insights from patient reviews, clinician notes, and medical research. This study evaluates the effectiveness of three NLP models - Bidirectional Encoder Representations from Transformers (BERT), Valence Aware Dictionary and sEntiment Reasoner (VADER), and Flair - in analyzing patient sentiment from physician reviews. A total of 1,486 reviews of 30 pain management specialists in Atlanta, GA, were collected from Healthgrades, with sentiment scores derived from each model and compared to patient-provided numerical ratings.

Statistical analyses, including pairwise t-tests, Pearson correlation, and logistic regression, were conducted to assess each model’s performance. Results showed significant differences among models (*P* < 0.05), with Flair demonstrating the highest correlation with patient ratings (*r* = 0.80), followed by BERT (*r* = 0.74) and VADER (*r *= 0.59). Logistic regression analysis further supported Flair's superior predictive accuracy.

These findings highlight the potential of sentiment analysis in healthcare, offering an objective lens to interpret subjective patient experiences. Future research should focus on refining NLP models for medical contexts, integrating multimodal sentiment analysis, and addressing ethical considerations in patient data handling. By leveraging sentiment analysis, healthcare systems may improve patient satisfaction assessment, identify early signs of mental health concerns, and reduce documentation bias.

While the results are promising, this study is limited by its retrospective design, single geographic region, and reliance on publicly available online reviews, which may not reflect the broader patient population or clinical encounters. Real-world validation in diverse settings and prospective studies is necessary to confirm the clinical applicability of these models.

## Introduction

Natural language processing (NLP) is increasingly being integrated into healthcare to help analyze the vast quantities of unstructured text generated through clinical documentation, patient reviews, and electronic health records (EHRs) [1-4]. One of the most promising applications of NLP is sentiment analysis, which involves identifying and quantifying emotional tone, polarity, and subjective attitudes within text. In the healthcare domain, sentiment analysis has been used to assess patient satisfaction, improve administrative efficiency by automating coding for billing and processing insurance claims, monitor mental health trends, evaluate clinician well-being through internal communications, and identify biases in recommendation letters and training materials [5-13]. These capabilities are critical as healthcare systems emphasize patient-centered outcomes and data-driven approaches to quality improvement.

Sentiment analysis methods have evolved considerably over the past decade. Early lexicon-based tools assigned sentiment scores based on predefined word lists but often struggled with contextual nuances and sarcasm. Machine learning (ML) methods introduced statistical learning from labeled data, improving classification accuracy, while deep learning models, such as Long Short-Term Memory (LSTM) networks, enabled better contextual understanding. Most recently, transformer-based models such as Bidirectional Encoder Representations from Transformers (BERT) have leveraged attention mechanisms to achieve state-of-the-art performance across a wide range of NLP tasks. Alongside these, user-friendly tools such as Valence Aware Dictionary and sEntiment Reasoner (VADER) and Flair have emerged as accessible, open-source solutions for sentiment classification, offering varying levels of complexity and domain adaptability [8-10].

In this study, we evaluate three representative sentiment analysis models - VADER, BERT, and Flair - selected to reflect distinct NLP paradigms. VADER is a lexicon and rule-based model specifically designed for social media and short-text analysis, providing a lightweight benchmark for sentiment scoring. BERT is a deep, transformer-based model pre-trained on large text corpora and fine-tuned for sentiment analysis, representing the most advanced paradigm in contextual language understanding. Flair, built on character-level embeddings and recurrent neural networks, offers a hybrid architecture that balances performance with simplicity and multilingual support. This diversity allows for meaningful comparison of approaches ranging from rule-based heuristics to state-of-the-art deep learning. The objective of this study is to compare the performance of three NLP models: VADER, BERT, and Flair, in analyzing sentiment from patient reviews of pain management physicians, and to assess how well their sentiment scores correlate with actual patient-provided star ratings.

Using 1,486 publicly available online reviews of 30 pain management physicians in Atlanta, GA, collected from Healthgrades, we compare the sentiment scores generated by each model with the corresponding numerical star ratings provided by the same reviewer. We hypothesize that there will be a significant difference in the correlation between each model’s sentiment output and the patient-provided rating, allowing us to evaluate their relative effectiveness in this real-world healthcare setting.

By assessing how well each model aligns with patient-reported satisfaction, this study contributes to a growing body of work focused on applying NLP tools to healthcare feedback. Our findings may inform future use of sentiment analysis in quality monitoring, patient engagement, and digital health research, particularly in domains where structured patient experience data are limited or absent.

## Materials and methods

Data procurement

Physician reviews were obtained from Healthgrades, a publicly available physician review website [14]. A search was conducted for pain management physicians in Atlanta, GA. To ensure that only licensed physicians were included, only Medical Doctors (MDs) and Doctors of Osteopathic Medicine (DOs) specializing in pain management were considered.

To maintain an unbiased selection process, the first 30 pain medicine physicians who met the inclusion criteria were selected from a randomized, unsorted list. The inclusion criteria for this study were: (1) physicians practicing pain medicine and (2) physicians located within a 20-mile radius of Atlanta, GA. One of the authors accessed the Healthgrades website and applied these criteria to generate the physician list. From the resulting search, the first 30 eligible physicians were selected. Healthgrades produces a randomized, unsorted list that varies with each site visit, ensuring a non-biased and representative sample at the time of data collection. The sample was comprised of multiracial male and female physicians. A sample size of 30 physicians was utilized for this preliminary study to approximate a normal distribution, allowing for the extraction of statistically significant insights from a balanced representation of participants. Featured physicians, who may have paid for promotional placement, were excluded. Additionally, any non-physician providers and physicians with fewer than 20 written reviews were excluded. A total of 1,486 patient reviews were collected from the 30 selected physicians. Patient demographic information, including race and gender, was not disclosed on the Healthgrades website and, therefore, was not available for analysis.

For each eligible physician, the individual review section was accessed while keeping the default review sorting settings. Up to the first 100 reviews per physician were recorded. Along with each review, the corresponding star rating assigned by the reviewer was documented for further analysis.

This data collection approach aligns with previous methodologies used in physician review studies, such as the framework outlined by Cheng et al. [15]. Ethical considerations were addressed by using only publicly available, de-identified data that contained no protected health information. Reviews were collected in aggregate for research purposes, and no attempts were made to contact reviewers or physicians. As such, this study posed minimal risk and did not require oversight by an institutional review board (IRB).

Data structure

For the code below to properly read and manipulate the data, the dataset must have a column dedicated to the physician being reviewed, the review itself, and the rating associated with that review.

Model development

Certain packages must be installed to execute essential functions in the model. The pandas library is used to handle datasets, apply sentiment analysis, and store the results after conducting the analysis. The torch package is a fundamental machine learning framework for deep learning (PyTorch), and the BERT and Flair models must run properly.

The pipeline function from the transformers library provides pre-trained NLP models, loading a BERT-based sentiment analysis model. The specific BERT-based architecture used in this study is DistilBERT, a streamlined version of BERT fine-tuned for sentiment analysis on the SST-2 dataset. The motivation behind using DistilBERT was to balance accuracy with computation efficiency, as this model retains most of the functionality at a fraction of the power, making it practical to handle large datasets for sentiment analysis. The TextClassifier function from flair.models imports Flair’s sentiment analysis model from the Flair model library. This function works in tandem with the Sentence function from the flair.data library, which converts review text into Flair sentence objects that the Flair model can process. Finally, the SentimentIntensityAnalyzer from the vaderSentiment package calculates a compound sentiment score using the VADER methodology. Each of these models is a general-purpose tool, not specifically trained on healthcare-related text. They return an output between [-1, 1], and this study utilized statistical and visual methods to assess how well these scores aligned with the original patient ratings on a 1-5 scale.

If any package fails to load and returns an error, use pip in the Command Prompt to install the required packages before running the program. The general syntax for installing packages is provided in the following section.

Data Preparation

The script loads the dataset and, if necessary, drops any rows where a physician has no reviews as part of the data-cleaning process. A 'latin1' encoding is used to prevent encoding-related errors. The dataset used for this study did not require extensive data cleanup; however, larger datasets may require more cleanup and standardization that are not outlined in this model design.

Initialize Models

Each variable listed loads its respective sentiment model and stores it as vader, bert_classifier, and flair_classifier, respectively. These variables specifically invoke the sentiment models, as many of these models can perform multiple types of analysis.

Sentiment Analysis Function

The analyze_sentiment function processes a given review in plain English by first checking for content. If the review is empty, the function returns None. Otherwise, it proceeds with sentiment analysis using two approaches.

First, it applies the VADER sentiment analysis tool, specifically utilizing the compound score, which provides a weighted sentiment value ranging from -1 (most negative) to +1 (most positive).

Next, the function employs a BERT-based sentiment classifier to analyze the review. This model returns a dictionary containing two key pieces of information: a confidence score between 0 and 1, representing the model's certainty in its classification, and a predicted sentiment label, either *POSITIVE* or *NEGATIVE*, which helps determine the final sentiment outcome. For the full script, refer to the Appendix.

The Flair technique first converts the review into a Flair Sentence object. It then applies a sentiment model to the sentence variable and extracts a confidence score along with a corresponding classification of either *POSITIVE* or *NEGATIVE*, similar to the BERT process.

Apply Sentiment Analysis to the Dataset and Export Findings

The analyze_sentiment function is applied to each review in the dataset (df). The resulting sentiment scores are then stored in new columns titled "VADER_Score", "BERT_Score", and "Flair_Score". These updated results must be exported to a file to consolidate the original data with the newly generated sentiment scores; however, users may choose to export the data to a different file format or directly to a website, depending on their use case. For the full script, refer to the Appendix.

All modeling up to this point was conducted in Python (version 3.12.5) and involved calculating sentiment scores using the VADER, BERT, and Flair models (Appendix). 

Statistical analysis

All statistical analyses were done using RStudio (version 2024.04.2). The initial analysis included pairwise t-tests to compare each scoring methodology with the others. Subsequently, once significant differences were obtained, we performed a Pearson correlation analysis on the data to identify which scoring methodology correlated best with the original ratings. Additionally, a logistic regression was conducted to evaluate the ability of each model to properly categorize reviews as positive or negative. Reviews rated 1-3 were considered negative, and those rated 4-5 were considered positive, as this classification is considered best practice in previous studies [16]. Statistical significance was defined as any *P* < 0.05.

## Results

**Figure 1:**
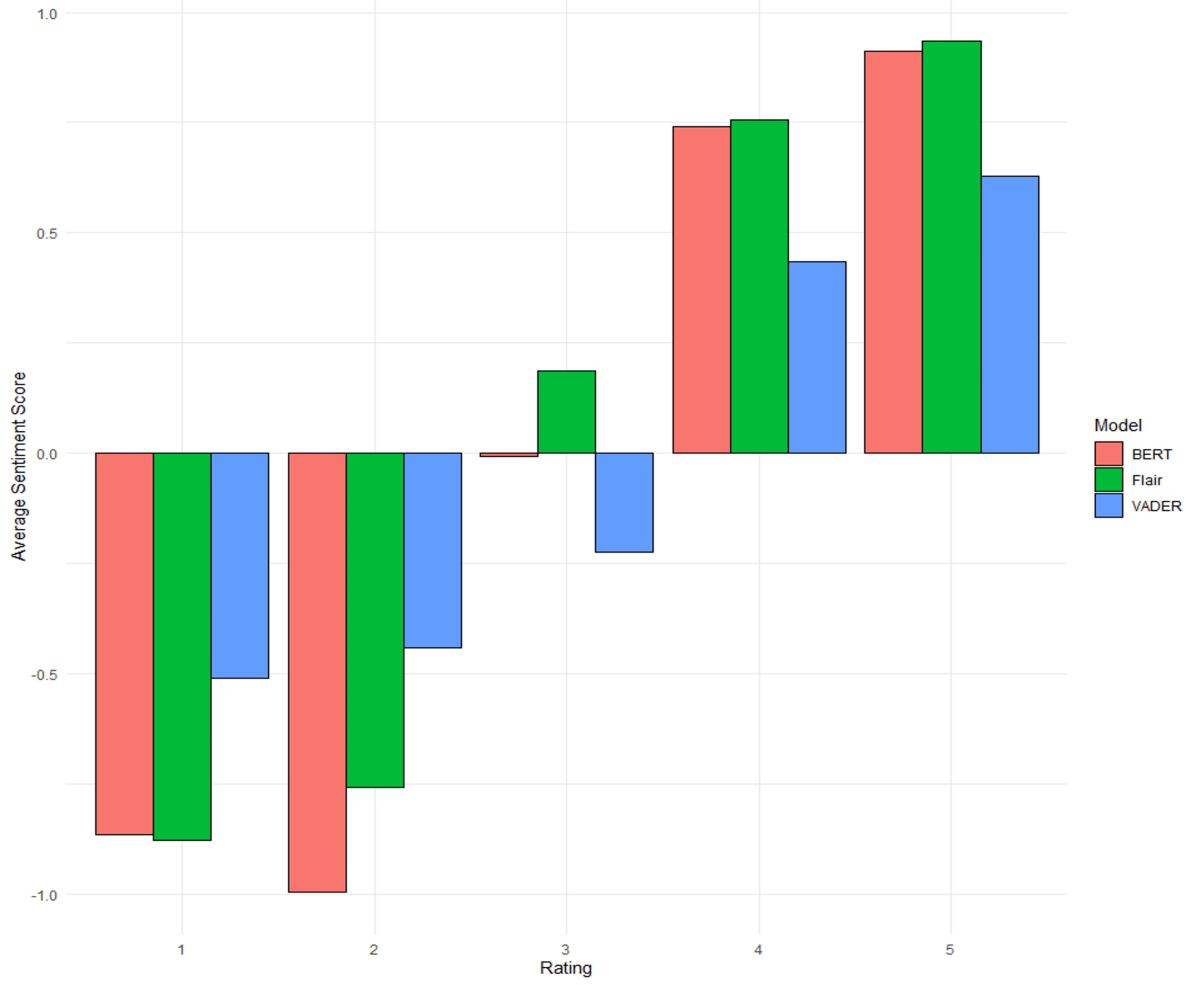
Average sentiment scores provided by natural language processing models based on patient ratings. VADER, Valence Aware Dictionary and sEntiment Reasoner; BERT, Bidirectional Encoder Representations from Transformers

**Figure 2:**
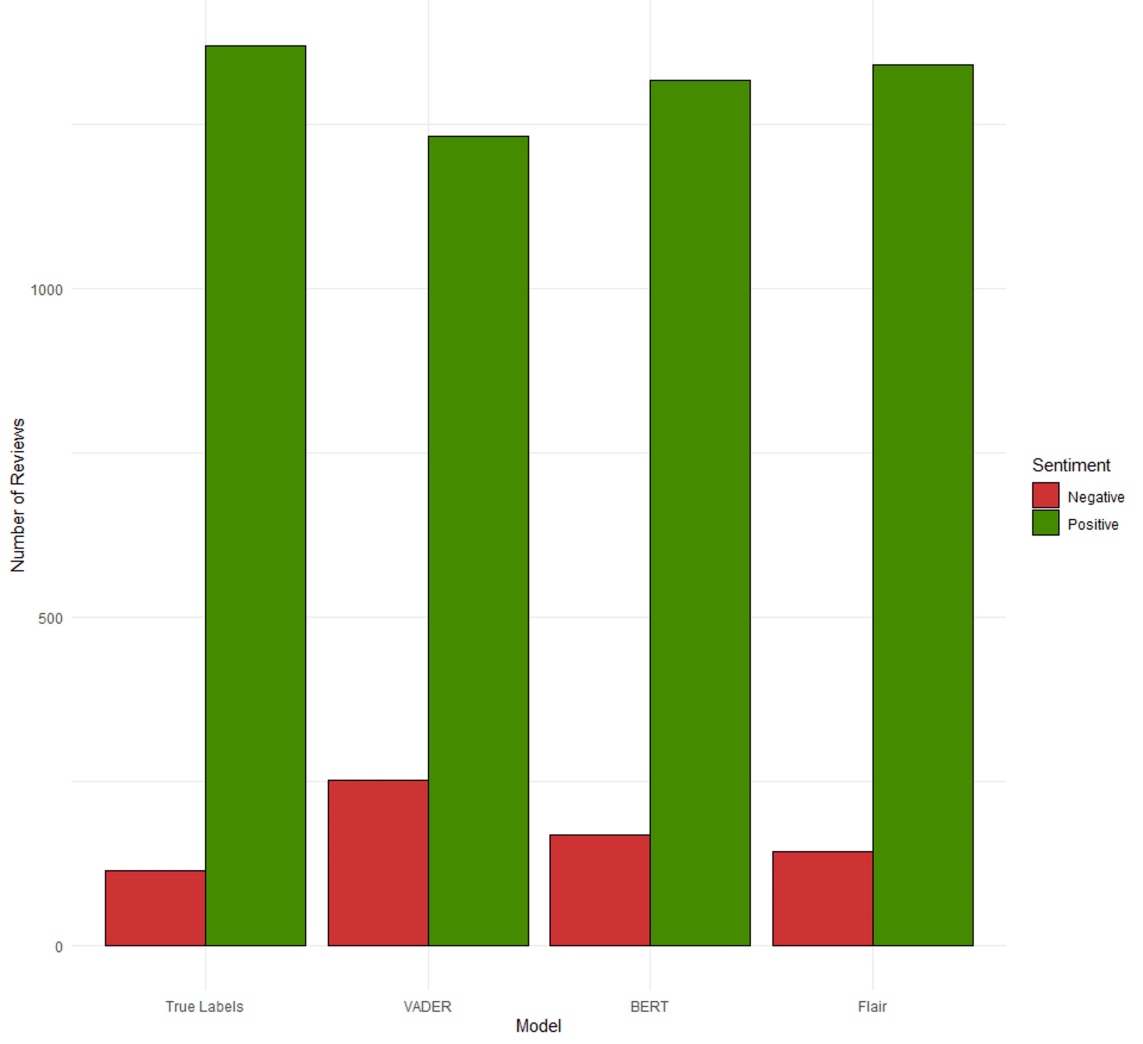
Frequency of positive and negative reviews stratified by model. VADER, Valence Aware Dictionary and sEntiment Reasoner; BERT, Bidirectional Encoder Representations from Transformers

Statistical analysis of VADER, BERT, and Flair sentiment analysis performance yielded various insights. Figure 1 provides a visual representation of the average scores returned by each sentiment model. The figure highlights both Flair and BERT models’ ability to capture sentiment in text compared to VADER. The model is particularly well-trained in handling extremes, which is expected as extreme values are often associated with definitive negative or positive diction, as opposed to more ambiguous sentiment expressed when a patient rates their physician as a 3 rating. Moreover, Figure 2 illustrates the distribution of positive and negative patient reviews alongside their classification by various NLP models. Notably, both Flair and BERT more accurately aligned with the true sentiment of the reviews compared to VADER, which, while adequate, demonstrated relatively lower performance compared to the other two models.

Each of these three models yielded varying results from one another, with statistically significant differences. The *P*-values for each set of comparisons are as follows: (1) VADER versus BERT (*P* = 2.2e-16), (2) VADER versus Flair (*P* = 2.2e-16), and (3) BERT versus Flair (*P* = 0.02413). This highlights that although these three models achieve similar functions, their sentiment analysis efficacy varies in performance.

First, a correlation analysis was performed to evaluate the strength of the relationship between the sentiment analysis scores to the original, patient-provided numerical reviews. Based on our analysis, all three relationships were statistically significant. In comparing the strength of the correlation analyses, Flair demonstrated the strongest correlation with the ratings (*r* = 0.80), followed by BERT (*r* = 0.74), and finally, VADER with the weakest correlation (*r* = 0.59). While VADER yielded the lowest correlational strength, it still demonstrated a positive trend between sentiment analysis scores and patient-provided numerical reviews.

Additionally, a logistic regression was conducted to quantify the efficacy of each NLP model. To evaluate model performance, we randomly partitioned the dataset using a 70/30 train-test split with stratified sampling on the outcome variable (i.e., positive vs. negative sentiment). The training set was used to fit the logistic regression model, while the test set was used to calculate predictive accuracy and performance metrics. This validation split was performed using createDataPartition() in R, ensuring balanced class representation across the split. Table 1 captures the tool-specific β-coefficient from the logistic regression and its associated *P*-value.

**Table 1:** Statistical outcomes from linear regression. VADER, Valence Aware Dictionary and sEntiment Reasoner; BERT, Bidirectional Encoder Representations from Transformers

Tool	Estimate (β)	*P*-value
VADER	1.3102	0.000243
BERT	1.0498	0.000325
Flair	1.5042	<0.000001

The calculated accuracy of the logistic regression was 98.65% (F1 = 0.9153; receiver operating characteristic-area under the curve (ROC-AUC) = 0.9883), indicating that models correctly identified sentiment across approximately 98% of reviews. When stratified by tool, as substantiated by the correlation analysis, based on logistic regression coefficients, Flair offered the greatest predictive capacity of patient-provided numerical reviews. Table 2 further displays the accuracy breakdown by tool.

**Table 2:** Tool accuracy, F-statistic, and receiver operating characteristic-area under the curve (ROC-AUC). VADER, Valence Aware Dictionary and sEntiment Reasoner; BERT, Bidirectional Encoder Representations from Transformers

Tool	Accuracy	F1	ROC-AUC
VADER	88.6%	0.83	0.85
BERT	93.1%	0.89	0.91
Flair	95.3%	0.92	0.94

**Figure 3:**
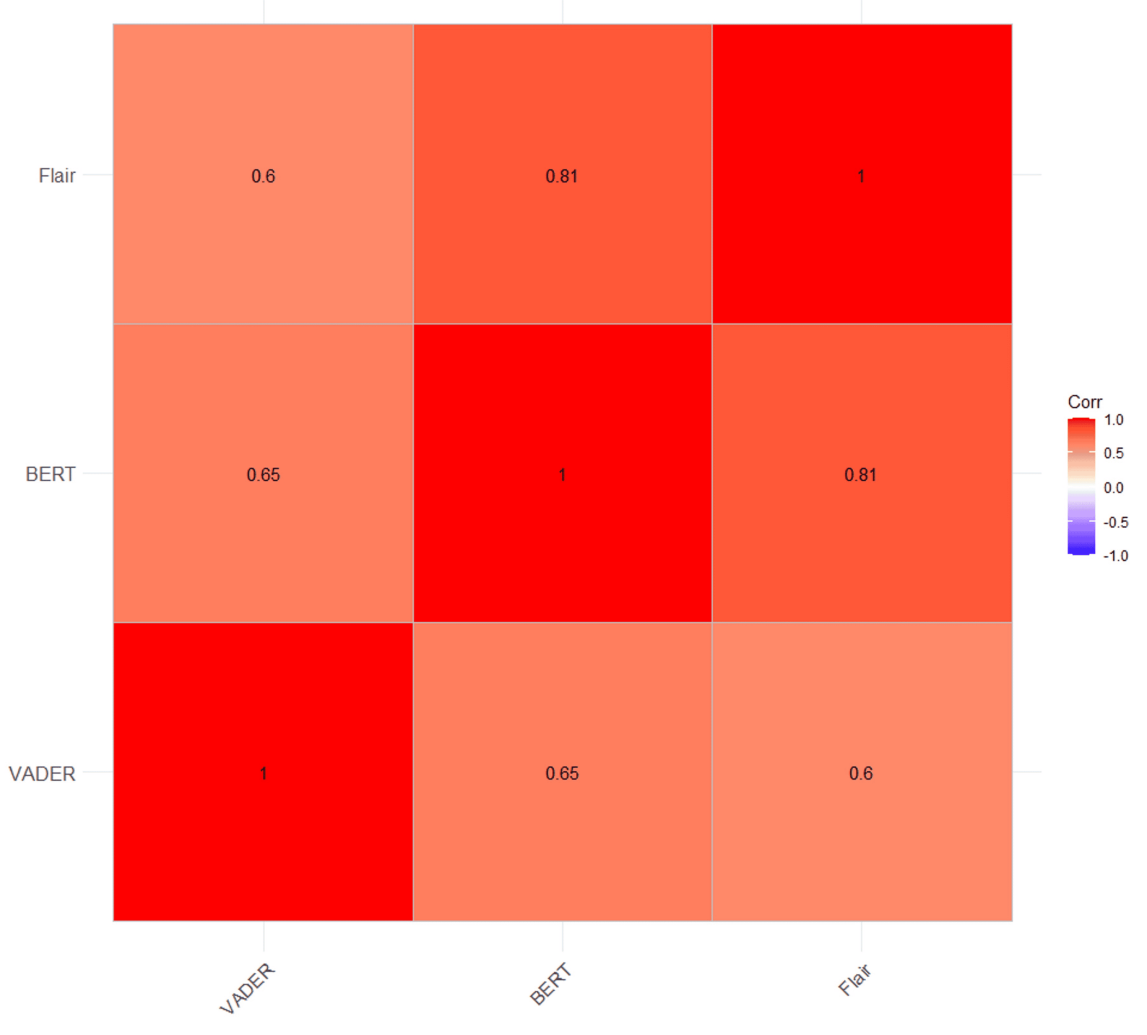
Correlation heatmap between sentiment models.

**Figure 4:**
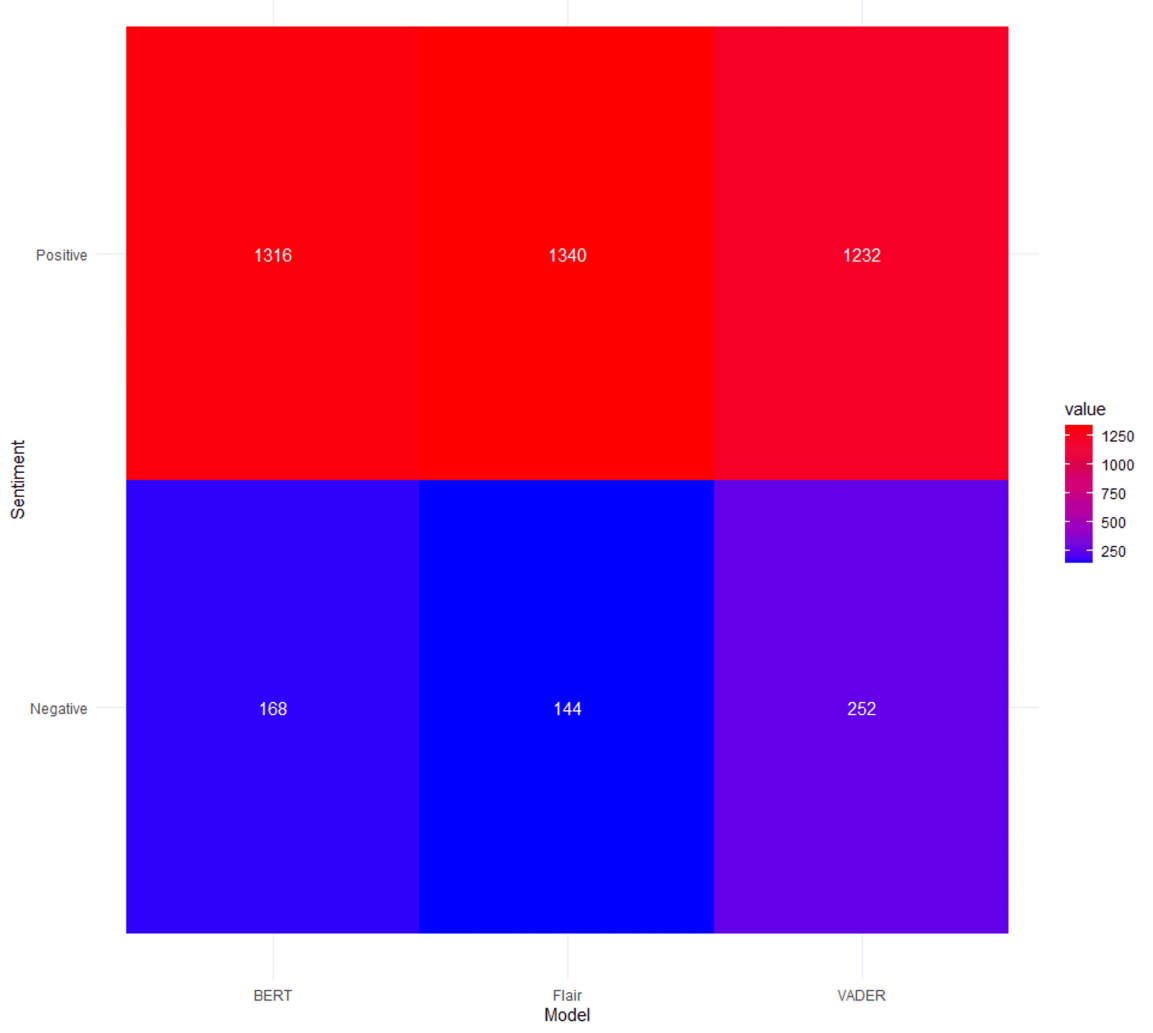
Agreement heatmap of positive and negative reviews stratified by model.

Figure 3 represents a correlation heatmap between the three NLP models, demonstrating that highly correlated scores indicate similarly behaving models. Additionally, Figure 4 presents an agreement heatmap, with the X-axis representing sentiment models (VADER, BERT, Flair) and the Y-axis divided into positive and negative reviews. Color intensity indicates the frequency of agreement among models on *Positive* versus *Negative* classifications. This analysis illustrates how higher agreement levels indicate that models have similar classification tendencies.

Ultimately, this data analysis demonstrates the ability to compare NLP model functioning based on correlation with true values, logistic regression values based on effectiveness of predictive ability, and tendency to categorize data into *Positive* or *Negative* classifications. Such a statistical analysis demonstrates a proof-of-concept that these NLP models can accurately read and interpret patient reviews, assigning a numerical value to otherwise subjective information. These preliminary results demonstrate positive potential, since this study employed only one dataset and three NLP models, and linear regression models require training with large datasets. Therefore, further training of statistical models and broader datasets is fundamentally required to establish significant findings.

## Discussion

In this study, we demonstrated the practical application of sentiment analysis to evaluate patient reviews of pain and spine anesthesiologists using real-world data. By comparing the performance of three distinct NLP models - VADER, BERT, and Flair - we provided a comprehensive analysis of how each model interprets sentiment in healthcare-related text. Notably, the Flair model achieved the strongest correlation with patient ratings (*r* = 0.80), suggesting that it is particularly well-suited for capturing nuanced emotional tone in patient-authored content. These findings support the growing utility of NLP-driven sentiment analysis as a tool for extracting meaningful insights from unstructured health communication.

A major strength of this work lies in its use of publicly available, real-world patient feedback, offering a scalable and transparent approach to evaluating sentiment models in healthcare. This enhances both ecological validity and relevance for deployment in patient experience monitoring systems. While our dataset offers a valuable real-world sample of patient feedback, the substantial skew toward 5-star reviews limited the opportunity to evaluate model performance across a balanced sentiment spectrum. Incorporating a more diverse distribution of reviews, including a greater proportion of neutral and negative feedback, would provide a more comprehensive assessment of classification accuracy across the full range of patient sentiment. We further employed multiple statistical techniques, including Pearson correlation, logistic regression, and ROC-AUC, to rigorously compare model performance. The inclusion of open-source Python code also supports reproducibility and invites further innovation.

Importantly, we selected three models representing distinct paradigms: VADER (rule-based), BERT (transformer-based), and Flair (hybrid RNN-based with character-level embeddings). This comparative design allowed us to benchmark the strengths and limitations of each tool without the need for extensive customization, reflecting realistic constraints in many healthcare settings. Our results reaffirm that transformer-based models like BERT and Flair offer notable advantages in handling semantic nuance, syntactic variation, and polysemy-critical capabilities in the interpretation of healthcare language [17].

While the findings are encouraging, future research should expand upon this work in several key areas. First, although our dataset of 1,486 reviews from 30 pain specialists provided sufficient statistical power for initial comparisons, broader inclusion of specialties, geographic regions, and healthcare settings would improve generalizability. Second, we did not include domain-specific transformer models such as ClinicalBERT or BioBERT. Prior work has shown that these models outperform general-purpose alternatives in biomedical tasks. For instance, BioBERT has demonstrated F1-scores of 0.89 for named entity recognition, and ClinicalBERT has been reported to achieve superior accuracy in sentiment classification involving clinical narratives compared to baseline BERT models [17,18]. More recently, a study comparing Bio+Clinical BERT to general-purpose BERT and convolutional neural network (CNN) models for patient drug-review sentiment classification found that Bio+Clinical BERT achieved up to an 11% improvement in macro-F1-score, further demonstrating the advantage of domain-specific adaptation in healthcare sentiment tasks [19]. Including such models in future studies would provide a more complete picture of state-of-the-art performance in clinical sentiment analysis.

Additionally, large language models (LLMs) such as GPT-4 continue to advance NLP performance across diverse domains, including sentiment classification. While still under evaluation in clinical contexts, these models hold promise for future applications and should be considered in ongoing benchmarking efforts [17].

We also emphasize the importance of ethical considerations when applying sentiment analysis in healthcare. While the use of publicly available, de-identified reviews aligns with ethical standards, future applications involving more sensitive data, such as electronic health record notes or patient communications, should adhere to strict data privacy regulations and be implemented within opt-in, transparent frameworks [20]. For example, while the idea of analyzing digital footprints for mental health monitoring is compelling, it must be balanced with patient autonomy, informed consent, and robust oversight.

Furthermore, this study highlights the need to address algorithmic and data-driven bias. Patient sentiment may reflect implicit biases related to physician demographics, such as race, gender, or language proficiency, which can skew perception and satisfaction independently of clinical performance. Studies have shown that minority and non-native English-speaking physicians are disproportionately rated lower by patients, despite similar outcomes [21]. Our models did not adjust for these confounders, which represents an area for refinement.

The sentiment models themselves may also encode societal biases from their training data. NLP systems trained on broad internet corpora can unintentionally reinforce inequities related to race, gender, or socioeconomic status [22]. Without debiasing techniques, such systems risk perpetuating disparities in healthcare delivery. Future work should incorporate model explainability methods, such as SHapley Additive exPlanations (SHAP) or Local Interpretable Model-agnostic Explanations (LIME), to audit for biased outputs and ensure fairness.

Demographic data about the reviewers and physicians were not available in our dataset, limiting subgroup analysis. Collecting such metadata in future studies will enable assessments of fairness, performance variability, and equity in sentiment interpretation across populations.

Finally, while sentiment analysis tools show potential for real-time feedback monitoring and predictive applications, such as identifying patients at risk for dissatisfaction, non-adherence, or readmission, clinical integration should prioritize interpretability, human oversight, and regulatory compliance. For example, models deployed in healthcare must comply with the Health Insurance Portability and Accountability Act (HIPAA) and support clinician validation of outputs before decisions are made [5,23].

In conclusion, this study illustrates the feasibility and value of using NLP-based sentiment analysis to interpret patient reviews in a healthcare context. By comparing distinct model architectures, employing robust statistical validation, and considering ethical and methodological challenges, we lay the groundwork for future use of sentiment tools in enhancing patient-centered care. With continued refinement, domain-specific benchmarking, and commitment to equity and transparency, sentiment analysis can play an integral role in improving healthcare delivery and amplifying the patient voice.

## Conclusions

This study demonstrates the potential utility of sentiment analysis for interpreting patient-generated feedback in healthcare. By evaluating three widely used NLP models - VADER, BERT, and Flair - on 1,486 patient reviews of pain management physicians, we found that transformer-based approaches, particularly Flair, exhibited stronger alignment with patient satisfaction ratings. Using Pearson correlation, logistic regression, and ROC-AUC, we quantitatively compared model performance, and visualizations such as heatmaps and score distributions further illustrated areas of model agreement and divergence. These findings support the feasibility of using sentiment analysis to translate subjective narratives into structured insights that can enhance patient experience monitoring.

However, our results should be interpreted within the context of the study’s exploratory design. The dataset was limited to one specialty and geographic region, and all models evaluated were general-purpose rather than fine-tuned for clinical language. As such, conclusions regarding clinical readiness should be made cautiously. Broader applications, such as real-time sentiment monitoring or integration into clinical decision-making, will require external validation using diverse, multi-site datasets, domain-specific models (e.g., ClinicalBERT or BioBERT), and human-annotated sentiment benchmarks to ensure accuracy and generalizability.

To ensure fairness, transparency, and real-world usefulness of sentiment analysis tools in healthcare, it is essential to involve key stakeholders early and meaningfully. This includes organizing co-design workshops with clinicians, patients, and ethicists to review and refine model outputs, creating patient advisory boards to guide consent and data use policies, and piloting tools in clinical environments to gather implementation feedback. An ethics oversight committee should be tasked with monitoring for bias, privacy risks, and fairness, while a user-facing feedback mechanism should enable error reporting and ongoing model improvement. Through continued refinement, prospective validation, and stakeholder engagement, sentiment analysis can evolve into a technically sound, ethically grounded, and clinically relevant tool for supporting patient-centered care.

## Figures and Tables

**Figure 5:**
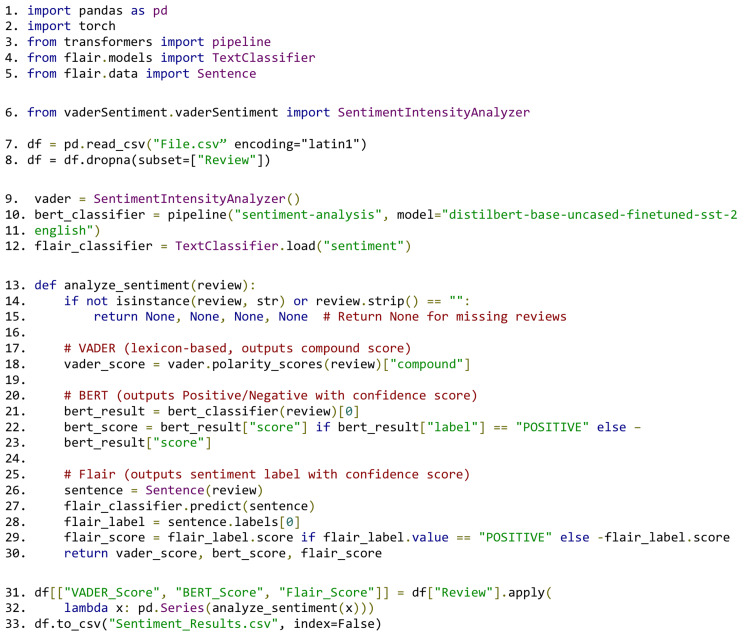
Code utilized analyze sentiment analysis via BERT, VADER, and Flair. The program was developed in Python version 3.12 and calculates sentiment scores using the VADER, BERT, and Flair sentiment models. GitHub link for code access: https://github.com/sjoseph30/SentimentAnalysis/blob/main/Sentiment%20Model.

## Appendices

Appendix
